# A feasibility study of distress screening with psychometric evaluation and referral of cancer patients

**DOI:** 10.1038/s41598-025-94538-5

**Published:** 2025-03-26

**Authors:** Daniel Anderson, Panagiotis J. Vlachostergios, Lilly Simpson, Susanna Schuster Bruce, Niall Fitzpatrick, Jacqueline Connell, Eleftherios Christodoulis, Konstantinos Kamposioras

**Affiliations:** 1 Department of Psycho-Oncology, The Christie NHS Foundation Trust, Manchester, UK; 2 Department of Medical Oncology, IASO Thessalias Hospital, Larissa, Greece; 3 Division of Hematology and Medical Oncology, Department of Medicine, Weill Cornell Medicine, New York, USA; 4 Department of Medical Oncology, The Christie NHS Foundation Trust, Manchester, UK; 5 Division of Cancer Sciences, Faculty of Biology, Medicine and Health The University of Manchester, Manchester, UK

**Keywords:** Cancer, Screening, Anxiety, Depression, Psycho-oncology, Cancer, Psychology, Health care, Medical research, Oncology

## Abstract

To assess for associations of known psychometric scales GAD-7, PHQ-9, pc-PTSD-5 with demographics and clinical characteristics of cancer patients as well as identify their value in screening for distress and in guiding Psycho-Oncology evaluation. This prospective feasibility study employed three psychological testing questionnaires, specifically the GAD-7, PHQ-9, and PC-PTSD-5, for the purpose of distress screening. Patients with a diagnosis of colorectal cancer who scored highly on at least one psychometric scale (defined in previous studies of non-cancer populations as a GAD-7 score of 10 or above, a PHQ-9 score of 10 or above, or a PC-PTSD-5 score of 4 or above) were offered a referral for further assessment in the Psycho-Oncology service and triaged via a semi-structured interview. The relationship between patients’ demographics and clinical characteristics and scores and outcomes was evaluated using the chi-square test. Fifty-four patients (30 females) of median age 60 years (range 36–81) were evaluated in the study. Thirty-four patients (63%) scored high on GAD-7, 40 (74%) on PHQ-9, and 8 (15%) on PC-PTSD-5 scales, respectively. Twenty-nine out of the 54 patients who underwent initial assessment with the psychometric scales (53.7%) accepted to be referred to the Psycho-Oncology service and were triaged via semi-structured interview while the rest 25/54 (46.3%) patients declined further assessment. Twenty-two of the patients who were interviewed (76%) required further specialist Psycho-Oncology intervention and the rest were signposted to community services. Patients younger than 65 years of age were more likely to score high according to the GAD-7 tool (*p* = 0.036). White Caucasian patients tended to score higher in the PHQ-9 questionnaire compared to non-white ones (*p* = 0.07). Prior history of mental disorder was significantly associated with higher scores in both GAD-7 (*p* = 0.041) and PC-PTSD-5 tools (*p* = 0.016). Patients who accepted a referral for psycho-oncology intervention demonstrated statistically elevated levels of anxiety on GAD-7 (*p* = 0.007) and diminished levels of depression on PHQ-9 (*p* = 0.042) compared to those who declined the referral. A clinical pathway involving a stepwise approach of psychometric scale evaluation and semi-structured interview can appropriately identify cancer patients with distress requiring further psychological support.

## Introduction

Cancer diagnosis and treatment is related with increased levels of psychological distress, including anxiety, depression and combination of mood changes up to the 40% of cases^1^. Throughout the course of the COVID-19 pandemic, these alterations became increasingly pronounced, affecting both the general population and at-risk groups, including cancer patients^2,3^. However, identifying patients at risk and referring them to the appropriate services for expert advice and management remains a challenge. Particularly for cancer patients offering timely psychological support remains an unmet need due to limited hospital and community psycho-oncology services, clinician time constraints, and poor implementation of screening strategies^4^.

During the pandemic, the National Institute for Health and Care Excellence (NICE) recommended several measures to minimise cancer patient’s exposure to hospital environment and adjusted treatment strategies resulting in some cases, in postponement of non-vital cancer therapies^5^.

At the start of the COVID-19 pandemic, we reported that urgent deviation from standard cancer care, coupled with personal and social restrictions nationally, had a significant psychological impact on both cancer patients and their families^6^. We showed that there were patients needing more support but the lack of a discrete pathway for those scoring high in screening tools was missing^6^.

Expanding on our initial observations, the Psychological Impact of COVID-19 on Patients with Solid Malignancies (PICo-SM) Study was launched in April 2021. This single-institution study aimed to evaluate the levels of anxiety, depression, post-traumatic stress amongst patients with cancer during the COVID-19 pandemic. One of the secondary aims of this project was to evaluate the use of psychometric scales Generalised Anxiety Disorder Assessment (GAD-7), Patient Health Questionnaire-9 (PHQ-9) and Primary Care Post-traumatic Stress Disorder Screen (PC-PTSD-5)] as a screening tool for further psychiatric and psychological patient assessment. Another secondary aim was to assess for associations of known psychometric scales GAD-7, PHQ-9, pc-PTSD-5 with demographics and clinical characteristics of cancer patients.

The Psycho-Oncology department at our institution is a special service consisting of mental health nurses, psychology, psychiatry, and counselling. The service provides level 3 & 4 psychological and psychiatric assessment and intervention as previously described by the NICE Stepped Care Model^7^.

Herein we present the outcomes of our structured referral pathway model for patients diagnosed with cancer who scored above predetermined cut-off thresholds and were then referred to the Psycho-Oncology service for further assessment and management. There are various psychometric tools to detect distress among diverse populations, including cancer patients. Those mostly studied and individually validated include GAD-7, PHQ-9, and PC-PTSD-5 scales^8–10^. These distress screeners, particularly GAD-7 and PHQ-9 at 3 and 6 months may predict the need and to some degree the intention of cancer patients to seek psychosocial support^11^. Other psychometric scales including Hospital Anxiety and Depression Scale (HADS), the 12-item Short Form Health Survey (SF-12) quality of life (mental and physical), and the Distress Thermometer (DT) may also have an impact on the use of psychological care in the inpatient or outpatient cancer care settings^12,13^.

Recent evidence suggests that lowering cut-off scores, particularly for GAD-7 scale from 10 to 8 could enhance diagnostic accuracy^14^. However, lowering the cutoff for certain or all scales could significantly increase the sensitivity of these analyses thereby running the risk of overflowing psycho-oncology services with many false-positive cases. A more reasonable approach which however requires additional validation studies could be the use of abbreviated versions of the Work and Social Adjustment Scale (WSAS), PHQ-9 and GAD-7 which were correlated 0.95 with their original counterparts and could reduce patient burden with nearly 50%^15^.

Our work further expands knowledge in the field by not only combining all three major scales, including GAD-7, PHQ-9, and PC-PTSD-5 and their associations with patient demographics and clinical characteristics but also by integrating them into a structured clinical pathway in daily practice, with reporting of specific measurable outcomes.

## Methods

**Fig. 1:**
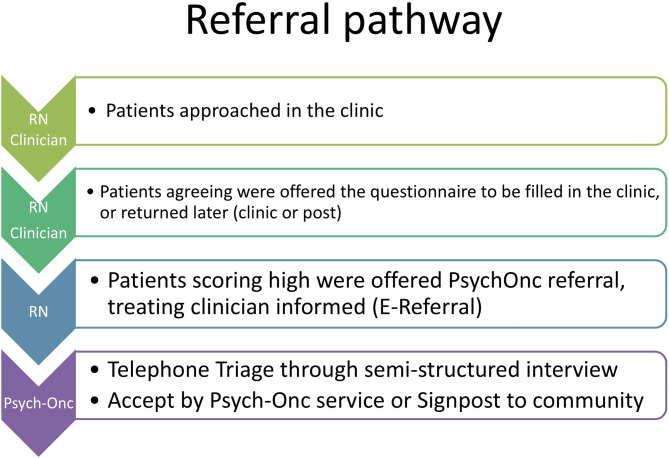
Algorithm of the clinical referral pathway. RN: research nurse.

Participants eligible for this feasibility study included those of age, with a diagnosis of colorectal cancer who were able to fully comprehend the patient information sheet for PICo-SM study. PICO-SM (IRAS Project ID − 292413) is a single centre longitudinal prospective study that included patients with diagnosed colorectal cancer attending the specialized lower gastrointestinal cancer clinics at the Christie NHS Foundation Trust (Manchester, UK)^16^. Patients included in this feasibility study were identified through a review of the list of clinics and, were recruited in person or remotely (via telephone or video consultations) (Fig. 1). Two distinct cohorts of patients were invited to participate in the study and each cohort was followed up after 6 months (T1 and T2). The first cohort was recruited between 7 and 28 April 2021 and the second cohort between 6 December 2021 and 21 February 2022. These were patients either on follow-up or undergoing active treatment with curative or palliative intent. This study aimed to provide assessment and support irrespective of the actual phase of treatment or/and monitoring.

Patients were asked to complete a survey which included the three psychometric scales: Generalized Anxiety Disorder scale (GAD-7) for anxiety^17^, Patient Health Questionnaire-9 (PHQ-9) for depression^18^, Primary Care Post-Traumatic Stress Disorder-5 (PC-PTSD-5) for probable PTSD^19^. Patients who scored high on at least one psychometric scale (GAD-7 score ≥ 10, PHQ-9 score ≥ 10, PC-PTSD-5 score ≥ 4) at either time point of the study were contacted by a member of the research nursing team who had previously completed a psychologically based communications training programme to increase their competence and confidence in virtual communication and offered referral for further assessment in the psycho-oncology service. (Fig. 1). An electronic referral to the psycho-oncology service was completed by the research nurse, and the responsible oncology consultant was notified of the patient’s high distress. The referred patients then triaged by clinical members of Psycho-Oncology service via a standardised semi-structured interview, with a decision made by the wider multi-disciplinary team relating to signposting to psychological services versus further interventions offered if deemed appropriate according to the stepped care model^7^.

Statistical analyses to assess the association between demographic, social, and clinical parameters including age (< 65 vs. ≥ 65 years), sex (male vs. female), ethnicity (white/Caucasian vs. Asian, Black or other), marital status (in relationship or married vs. single, divorced or widowed), children (no vs. yes), living alone (no vs. yes), patient’s perception of cancer status (controlled vs. progressive or uncertain) and history of mental disorder (no vs. yes, including any of the following conditions: anxiety, panic attacks, anorexia, bulimia, psychosis, depression, social phobia, attention-deficit disorder, obsessive-compulsive disorder, autism, post-traumatic stress disorder, alcohol abuse, drug abuse, bipolar disorder, personality disorder) and psychometric tool scores (GAD-7, PHQ-9, PTSD) were conducted with Chi-square test. Associations between dichotomous psychometric tool scores (low: <10 for GAD-7, PHQ-9 and < 4 for PTSD vs. high: ≥ 10 for GAD-7, PHQ-9 and ≥ 4 for PTSD) and outcomes of psycho-oncology evaluation (accepted, declined, signposted) were also examined with the Chi-square test. The level of statistical significance was set at 0.05.

## Results

A total of 216 patients with colorectal cancer from Cohort I and 96 patients from Cohort II participated in the study. The mean age of the patients was 65 ± 10 years, and 183 (58.6%) of them were male. A total of 54 patients (42 from the first and 12 from the second cohort), i.e. 17.4% of the total number of patients in both cohorts, scored high using the three psychometric scales. The mean age of patients was 60 years (range 36–81) with a slight female preponderance (56% versus 44%).

Twenty-one patients out of the 54 patients who underwent psychometric scale assessment (39%) indicated that their cancer was under control, while 33/54 (61%) perceived their condition as progressing or uncertain (Table 1). Approximately half of the patients (*n* = 26; 48%) reported a history of mental illness.

The median and mean (± SEM) scores on each psychometric scale for the entire cohort are presented in Table 1 for GAD-7, PHQ-9, and PC-PTSD-5, respectively. Thirty-four (63%) scored high on the GAD-7, 40 (74%) on the PHQ-9 and 8 (15%) on the PC-PTSD-5 scales. Twenty-two patients scored high on both GAD-7 and PHQ-9, 5 scored high on both GAD-7 and PTSD-5, and 5 scored high on both PHQ-9 and PTSD-5.

Individuals under 65 years of age were more significantly likely to score highly on GAD-7 scale (*p* = 0.036), and there was a trend towards a higher PHQ-9 score among white caucasian individuals compared to other ethnic groups (*p* = 0.07). Individuals with a history of mental disorder had significantly higher scores on both the GAD-7 and PTSD-5 scales (*p* = 0.041 and *p* = 0.016, respectively). Gender, marital status, presence of children, living alone and perception of cancer status were not associated with distress upon initial screening with the use of psychometric scales in this cohort (Table 1).

**Fig. 2:**
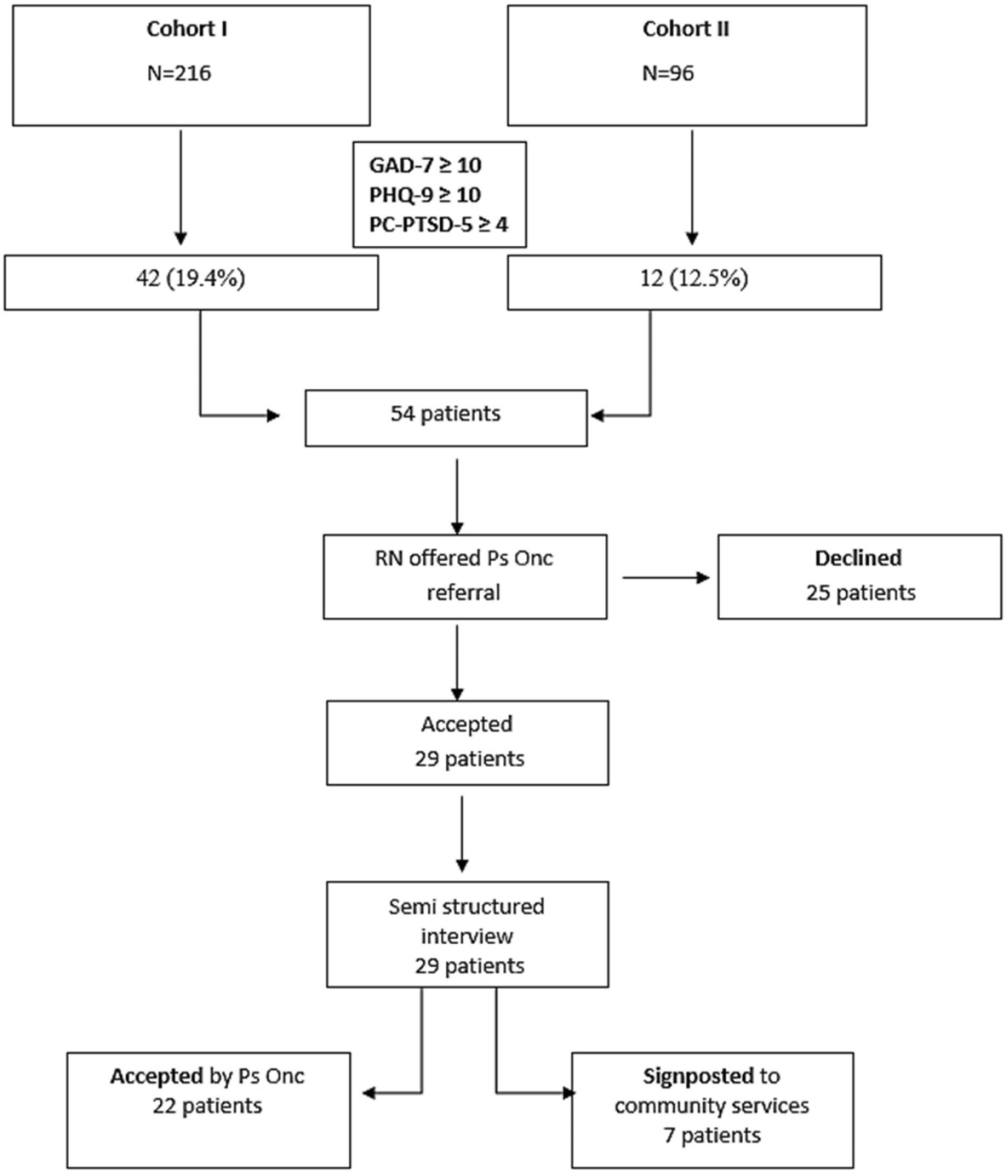
Flowchart of study patients.

Twenty-nine out of the 54 patients who underwent initial assessment with the psychometric scales (53.7%) accepted to be referred to the Psycho-Oncology service and were triaged via semi-structured interview while the rest 25/54 (46.3%) patients declined further assessment. Of the 29 patients who completed a triage assessment, 22 (76%) required further specialist Psycho-Oncology intervention and the rest were signposted to community services, creating there groups of interest (those who declined referral, those accepted by the psycho-oncology service and those signposted to community services) (Fig. 2).

Patients who accepted a psycho-oncology referral experienced higher anxiety levels on GAD-7 scale compared to those who declined (*p* = 0.007). Conversely, a higher mean and median PHQ-9 score was observed among patients who declined psycho-oncology referral compared to those who accepted or were signposted (*p* = 0.042) (Table 2). No statistically significant difference was observed in mean PTSD-5 scores (*p* = 0.36) between patients accepted by psycho-oncology for further management and those who were assessed and then discharged or declined referral.

**Table 1:** Demographics and clinical characteristics of patients.

Characteristic	N (%)	GAD-7		PHQ-9		PTSD	
Median (range)		11 (0–21)		13 (0–27)		0 (0–5)	
Mean (± SEM)		10.96 (± 5.75)		12.79 (± 6)		1.07 (± 1.55)	
Age (median)	60 (36–82)	Low (< 10)	High (≥ 10)	Low (< 10)	High (≥ 10)	Low (< 4)	High (≥ 4)
Age (mean)	60.89 (± 10.89)	9	25	8	26	27	7
< 65	34 (63%)	11	9	6	14	19	1
≥ 65	20 (37%)						
		**P = 0.036**		P = 0.600		P = 0.119	
Gender	54 (100%)	Low (< 10)	High (≥ 10)	Low (< 10)	High (≥ 10)	Low (< 4)	High (≥ 4)
Males	24 (44%)	9	15	5	19	21	3
Females	30 (56%)	11	19	9	21	25	5
		P = 0.950		P = 0.445		P = 0.668	
Ethnicity	54 (100%)	Low (< 10)	High (≥ 10)	Low (< 10)	High (≥ 10)	Low (< 4)	High (≥ 4)
White/Caucasian	46 (85%)	17	29	14	32	39	7
Other	8 (15%)	3	5	0	8	7	1
		P = 0.977		*P = 0.07*		P = 0.842	
Marital status	54 (100%)	Low (< 10)	High (≥ 10)	Low (< 10)	High (≥ 10)	Low (< 4)	High (≥ 4)
In relationship/Married Single/Divorced/Widowed	39 (72%)	14	25	12	27	32	7
	15 (28%)	6	9	2	13	14	1
		P = 0.780		P = 0.190		P = 0.286	
Children	54 (100%)	Low (< 10)	High (≥ 10)	Low (< 10)	High (≥ 10)	Low (< 4)	High (≥ 4)
Yes	43 (80%)	18	25	11	32	37	6
No	11 (20%)	2	9	3	8	9	2
		P = 0.147		P = 0.909		P = 0.725	
Living alone	54 (100%)	Low (< 10)	High (≥ 10)	Low (< 10)	High (≥ 10)	Low (< 4)	High (≥ 4)
Yes	10 (18.5%)	4	6	1	9	10	0
No	44 (81.5)	16	28	13	31	36	8
		P = 0.830		P = 0.203		P = 0.144	
Perception of cancer status	54 (100%)	Low (< 10)	High (≥ 10)	Low (< 10)	High (≥ 10)	Low (< 4)	High (≥ 4)
Controlled	21 (39%)	10	11	8	13	17	4
Progressive/Uncertain	33(61%)	10	23	6	27	29	4
		P = 0.199		P = 0.104		P = 0.485	
History of mental disorder	54 (100%)	Low (< 10)	High (≥ 10)	Low (< 10)	High (≥ 10)	Low (< 4)	High (≥ 4)
Yes	26 (48%)	6	20	7	19	19	7
No	28 (52%)	14	14	7	21	27	1
		**P = 0.041**		P = 0.872		**P = 0.016**	

Significant values are in bold.

**Table 2:** High-scoring patients across psychometric tools and clinical outcomes.

Scale	High score	Psych-Onc assessment			P value
		Accepted	Signposted	Declined	
GAD-7 N (%)	34 (63%)	11	2	21	**0.007**
Mean (± SEM)	14.59 (± 3.49)	14.38 (± 5.7)	16.25 (± 4.42)	*11.62 (*± *5.55)*	
Median (range)	14 (10–21)	19 (11–21)	*16 (12–21)*	12 (10–19)	
PHQ-9 N (%)	40 (74%)	17	9	14	**0.042**
Mean (± SEM)	15.32 (± 4.52)	*13.15 (*± 5.75)	13.33 (± 6.68)	*15.66 (*± *4.29)*	
Median (range)	14 (10–27)	13 (10–27)	12.5 (11–25)	14 (10–21)	
PC-PTSD-5 N (%)	8 (15%)	4	1	3	0.474
Mean (± SEM)	4.125 (± 0.35)	*4.25 (*± *0.5)*	*4 (*± *0)*	*4 (*± *0)*	
Median (range)	4 (4–5)	4 (4–5)	4 (4–4)	4 (0–4)	

Significant values are in bold.

Comparing sociodemographic characteristics between the 25 patients who declined further assessment and the rest 29 who accepted referral or/and signposted to community services, the former subgroup was of younger median age: 59 (36–92) vs. 64 (45–79) in those who accepted/signposted. The majority of the population was white/Caucasian in both subgroups (22 vs. 24). With respect to family status, the subgroup who declined assessment had numerically albeit not statistically significant more patients in relationship or married (21 vs. 18), with children (6 vs. 5), and less were living alone (3 vs. 7). Importantly, there was statistically significant difference in the perception of their cancer status, as the majority of those who declined assessment thought of their disease as being controlled (24/25) as opposed to only 15/29 in the subgroup who accepted/signposted (*p* < 0.001). In terms of personality stressors, while both groups shared the same number of patients with reported history of mental illnesses (*n* = 13), the subgroup who accepted/signposted were significantly more likely to suffer from self-reported depression (10/25) compared to those who declined (4/29) assessment (*p* = 0.028). No other statistically significant differences in sociodemographic factors were found between patients who declined and those who accepted referral or/and signposted to community services.

To investigate for an avoidance attitude among the patients who declined assessment compared to those who accepted or/and signposted to referral services we compared the number of those who answered yes to question 2: “Tried hard not to think about the event(s) or went out of your way to avoid situations that reminded you of the event(s)”. of the pc-PTSD-5 scale among the three subgroups (accepted, declined, signposted). Only 5/25 who declined had answered yes, as compared to 8/21 who accepted and 4/8 who signposted. Avoidance attitude as measured by question 2 did not differ significantly between patients who declined and those who accepted referral or/and signposted (*p* = 0.092).”

## Discussion

The prevalence of anxiety and depression in cancer patients is reported to have reached significant levels during the COVID-19 pandemic^3^. In this feasibility study, we aimed to develop a step-wise clinical pathway assessing the value of three psychometric scales, namely GAD-7, PHQ-9, and PC-PTSD-5 in identifying patients in psychological distress who would then benefit from a more thorough psychiatric referral for evaluation and appropriate psychological support. Patients triaged for Psycho-Oncology follow-up had significantly higher mean and median GAD-7 anxiety scores compared with those not accepted. The opposite was the case for PHQ-9 depression-screening scale. The precise reason for this phenomenon will require further investigation in a subsequent study, with the objective of elucidating the underlying reasons for the referral not being accepted.

To our knowledge this is one of the biggest prospective studies in colorectal cancer to longitudinally assess the psychological wellbeing of patients during the COVID pandemic and implement a referral pathway to address patients’ needs. The actual effectiveness of psychological screening alone has been debated^20^ and the semi-structured interview was used in conjunction to address any gaps in the screening process.

Whilst a number of patients were not accepted for Psycho-Oncology intervention, they warranted further lower-intensity intervention. As such, by effectively identifying patients with distress, screening via psychometric scales provides an opportunity for patients to receive an appropriate level of psychological support, as according to the stepped care model^7^.

These outcomes suggest that while psychometric scale tools were effective at identifying distress amongst cancer patients, the information gathered at triage, via semi-structured interview, helped significantly clinical decision making about specialist psycho-oncology support or signposting to the appropriate community service.

Prior to the COVID-19 pandemic, recommendations on the use of psychometric scales in cancer populations have been inconsistent. The American Society of Clinical Oncology (ASCO) guidelines recommend the use of PHQ-9 and GAD-7 for screening and assessment of depression and anxiety, respectively^21^. However, meta-analysis and a multi-centre study researching the validity of using HADS, GAD-7 and PHQ-9 respectively in cancer populations conclude these psychometric scales have limited screening performance compared with standardized diagnostic interview^22,23^. More recently, the European Society of Medical Oncology (ESMO) published their clinical guidelines on the screening for anxiety and depression in cancer patients^24^. Both PHQ-9 and GAD-7 can be used as screening tools using the thresholds we have used in our study for psycho-oncology referral.

A recently published systematic review evaluating the level of depression and anxiety during the coronavirus pandemic, suggests that the prevalence of distress among patients with cancer reached considerable levels during this time^3^. Nevertheless, there was substantial heterogeneity in the prevalence of anxiety and depression, dependent on which psychometric scales were used for screening. The study suggests that the cut-off values for PHQ-9 and GAD-7 anxiety were too low in the context of COVID-19 pandemic.

In accordance to previous studies^25^, almost half of the patients that scored high in the used scales declined referral to the appropriate service. Clover et al., in their study, reported that patient perception “they can manage on their own” and “the distress was not severe enough to be addressed through a tailored intervention” were the main reasons for declining support^25–27^. Although we did not systematically record the reason for refusal, anecdotally the most common response, when the patient was contacted by the research nurse to inform them and offer referral to the psycho-oncology service, was that they did not feel they were in any real distress. This was actually confirmed by comparing the perception of their cancer status, as the majority of those who declined assessment thought of their disease as being controlled as opposed to significantly fewer patients in the subgroup who accepted/signposted. Another explanation for this could be the higher frequency of self-reported depression and higher mean score of anxiety on GAD-7 scale in patients who accepted referral, whereas those who declined had higher median depression score on PHQ-9 scale despite underreporting depressive symptoms. While this may seem contradictory, it is important to consider that depressive symptoms may often run undiscovered, and these patients could be more reluctant to ask for evaluation. Avoidant coping may complicate psychological care seeking for a group of adults with cancer experiencing depressive symptoms and having a need for psychological care^28^. When we further investigated for an avoidance attitude among the patients who declined assessment compared to those who accepted or/and signposted using question 2: “Tried hard not to think about the event(s) or went out of your way to avoid situations that reminded you of the event(s)”. of the pc-PTSD-5 scale, we found no significant difference between patients who declined and those who accepted referral or/and signposted. Research is conducted to identify the determinants leading to variable acceptance rates to psycho-oncology support^29^ but challenges to approach difficult-to engage population have been reported^30^. Recommendation from the treating clinician could be a way to improve referral acceptance as confirmed by previous studies^31,32^.

In our study, the research nurses played a key role in contacting the patient for enrolment, but more importantly in informing the patient if they scored high enough to be referred to the psycho-oncology service. This was achieved through a training programme to increase their knowledge and confidence to contact the patient. Although this may not be the usual approach in other countries, the involvement of suitably trained allied health workers could be an important asset in this process.

Our study was limited by the small number of patients who were actually triaged. The exact reasons why patients declined referral for assessment (despite high psychometric scores) were not fully investigated. This cohort consisted of patients diagnosed with colorectal cancer, yet the applicability of this clinical pathway could include patients of any other cancer primary in line to what international guidelines recommend^21,24^. Although this study was carried out during the COVID pandemic, the proposed clinical pathway could be used at any time, as the need for psychological support for cancer patients remains, regardless of whether there are additional stressors on top of cancer.

Overall, overcoming barriers to operationalizing distress screening in cancer patients involves several strategies. Firstly, addressing individual characteristics, intervention specifics, implementation processes, and organizational contexts is recommended by the American Psychosocial Oncology Society and the Association of Oncology Social Work^33^. Secondly, implementing training programs for healthcare professionals, as in our study, can enhance knowledge and confidence in conducting distress screenings and referrals^34^. Third, fostering interdisciplinary collaboration among oncology teams, including nurses, psychologists, and oncologists, to ensure a comprehensive approach to distress management is key^35^. Additionally, identifying reasons for patient acceptance or refusal, as we attempted in our study, is also important to improve referral rates^26^. Finally, developing a structured process for screening, referral, and follow-up requires ensuring that resources are available to address identified distress, a situation that may be premature in low-resource settings^36^.

## Conclusion

This feasibility study supports the clinical utility of psychometric scales and semi structured interview used together to identify and triage cancer patients with distress that could benefit from an appropriate level of psychological support. Larger prospective studies are warranted to confirm whether this ‘screen and triage’ approach could be incorporated into a regional integrated care model for cancer patients, in order to improve access to appropriate levels of psychological support.

## Data Availability

The datasets used and/or analysed during the current study available from the corresponding author on reasonable request.
